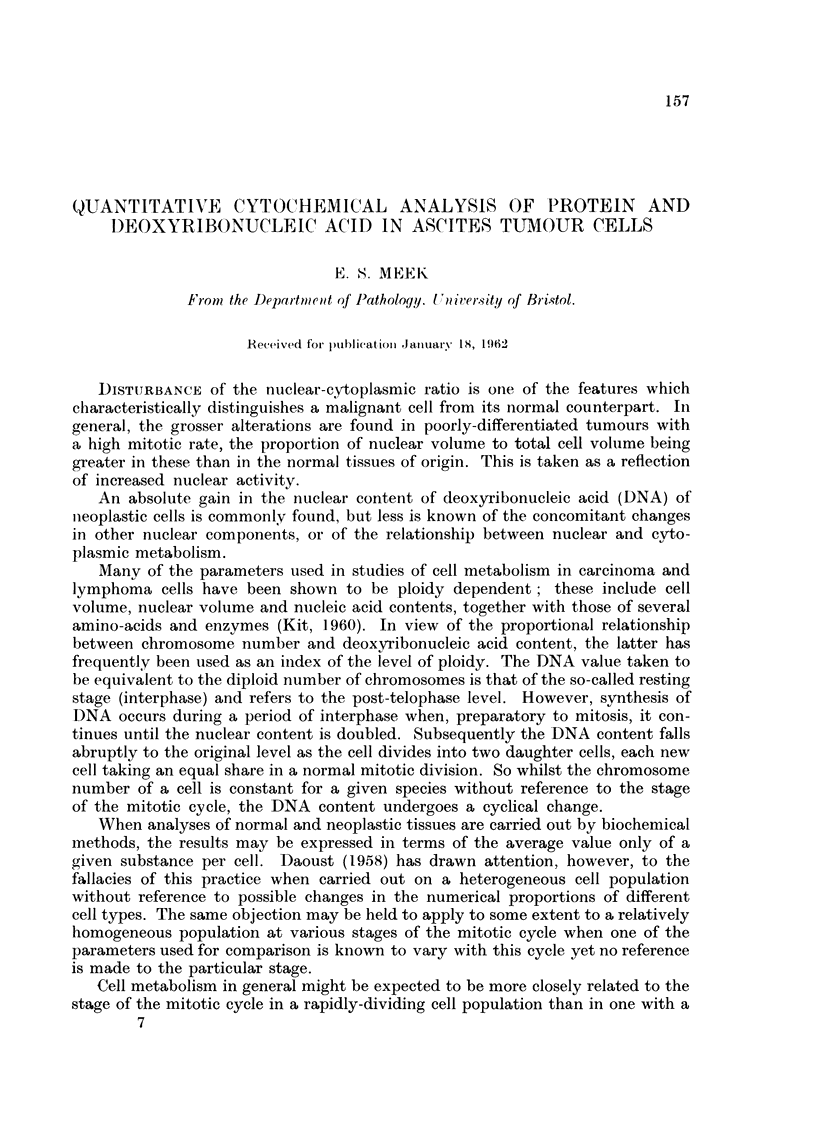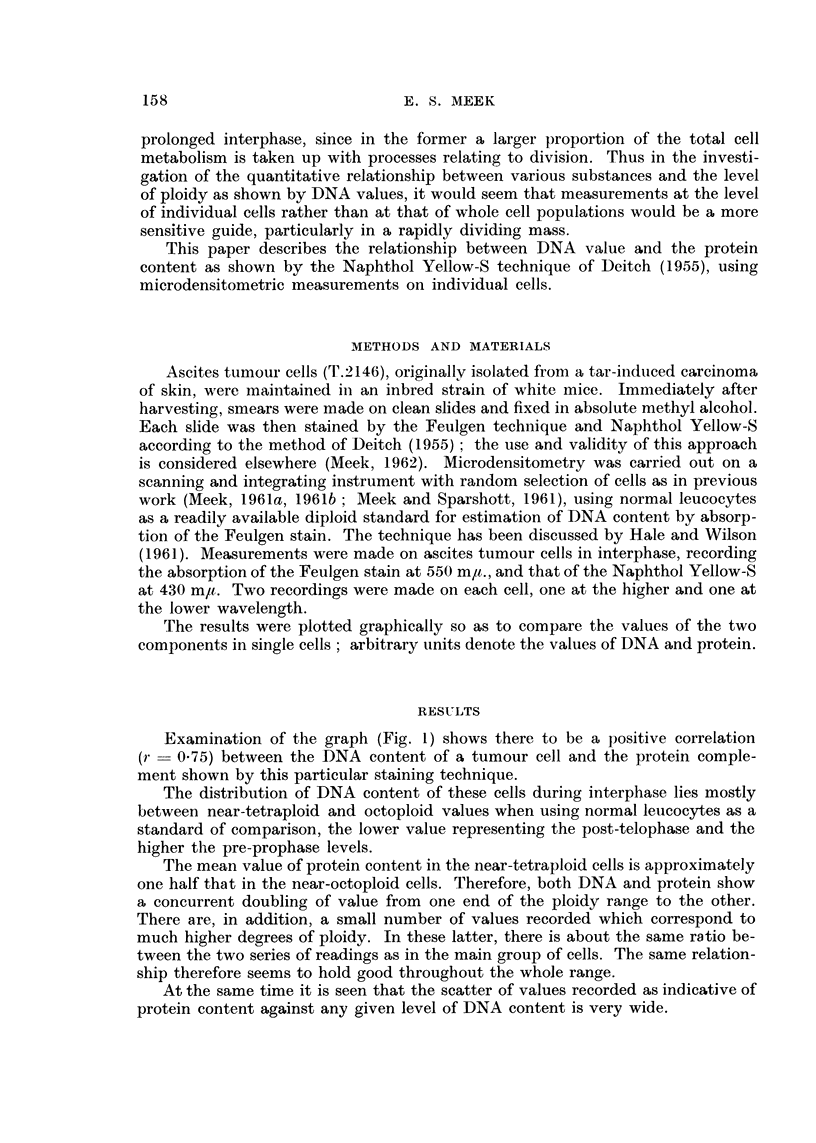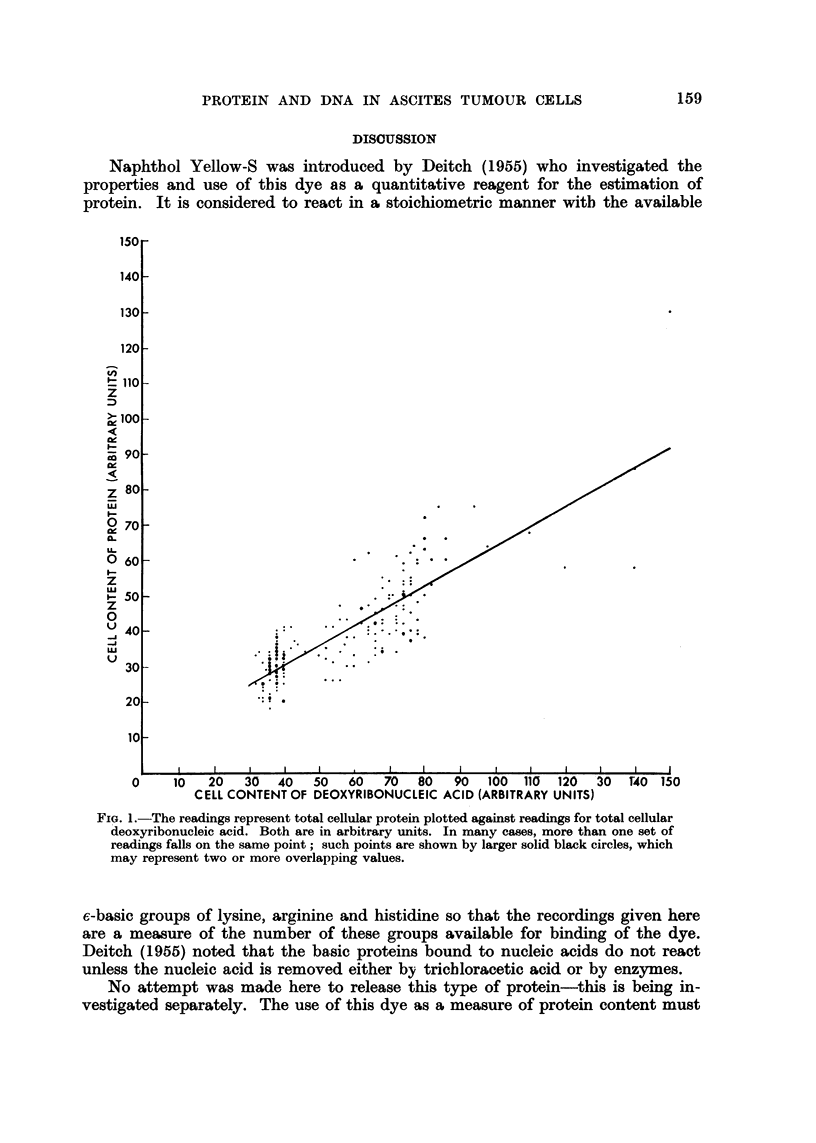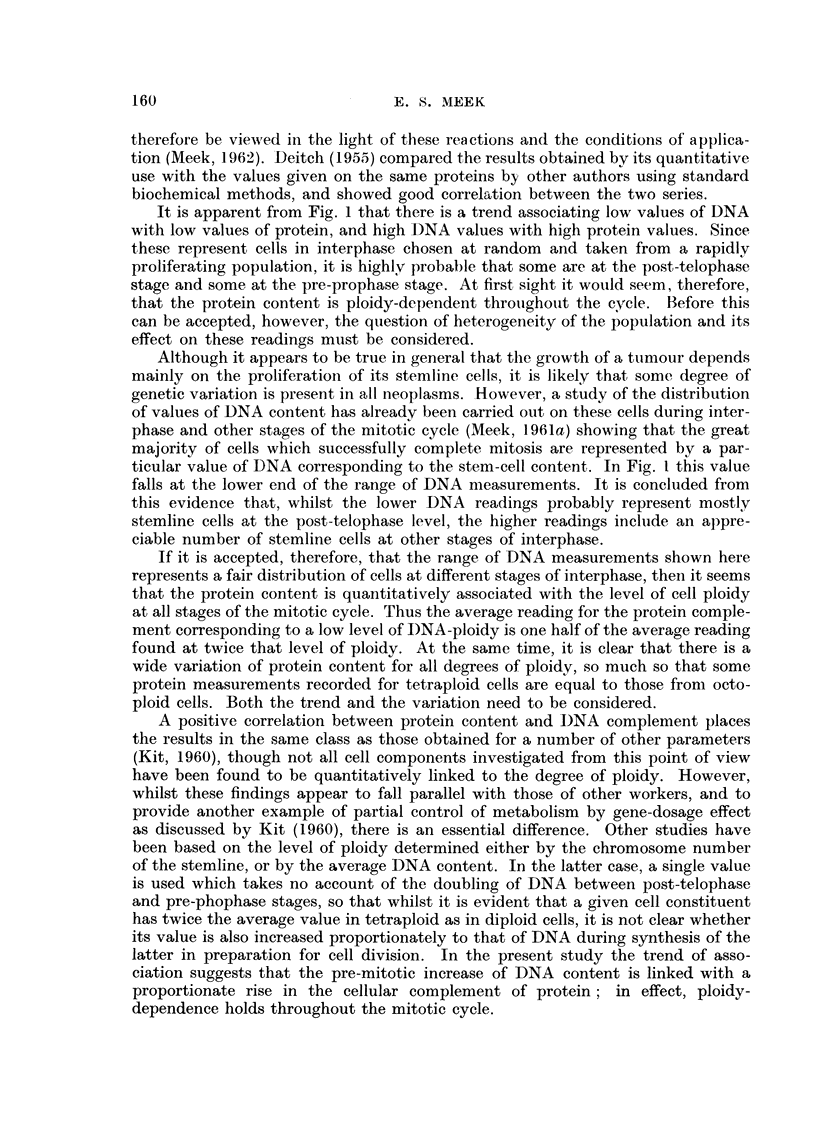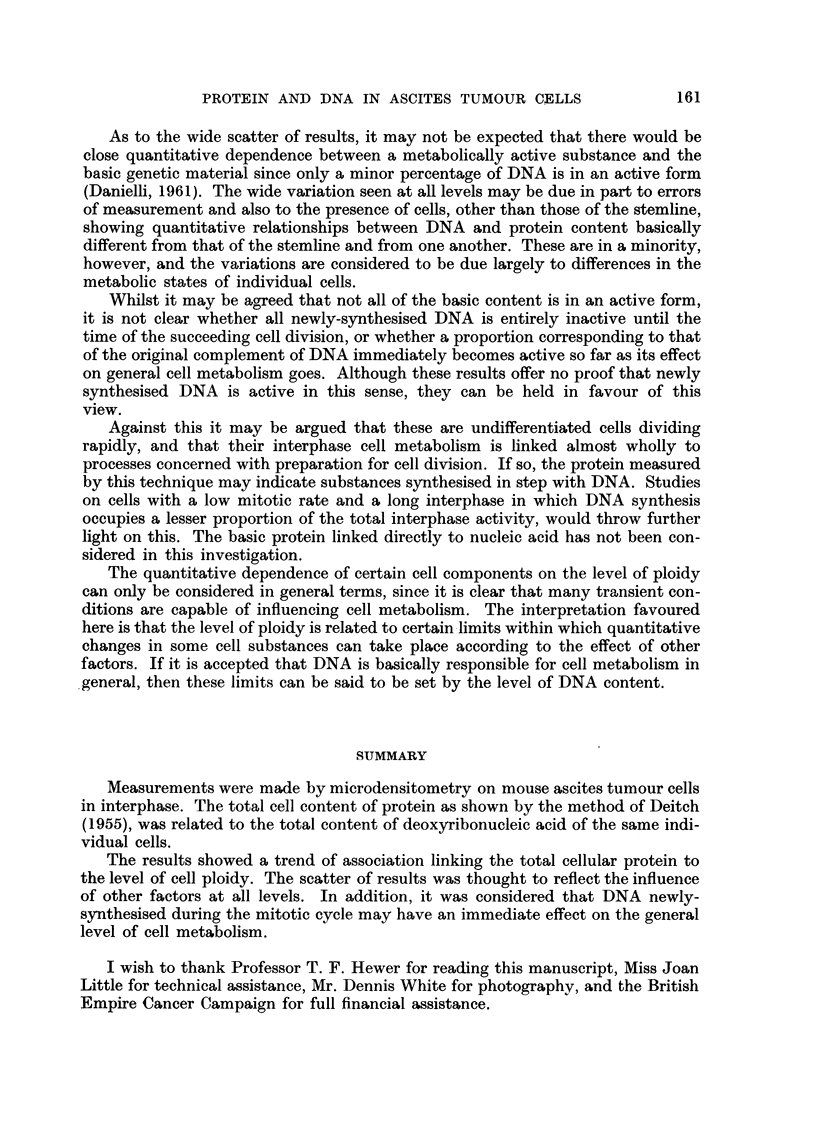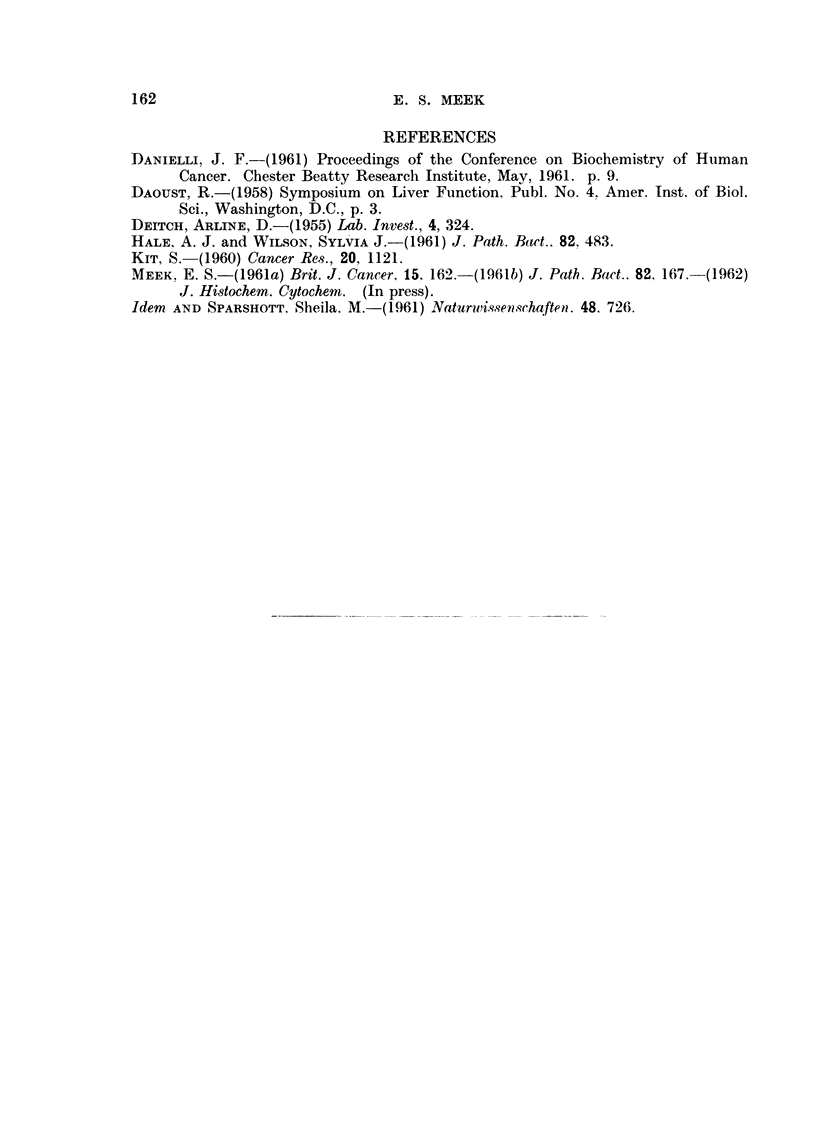# Quantitative Cytochemical Analysis of Protein and Deoxyribonucleic Acid in Ascites Tumour Cells

**DOI:** 10.1038/bjc.1962.16

**Published:** 1962-03

**Authors:** E. S. Meek


### QUANTITATIVE CYTOCHEMICAL ANALYSIS OF PROTEIN AND DEOXYRIBONUCLEIC ACID IN ASCITES TUMOUR CELLS

E. S. MEEK

*From the Department of Pathology, University of Bristol.*

Received for publication January 18, 1962

DISTURBANCE of the nuclear-cytoplasmic ratio is one of the features which characteristically distinguishes a malignant cell from its normal counterpart. In general, the grosser alterations are found in poorly-differentiated tumours with a high mitotic rate, the proportion of nuclear volume to total cell volume being greater in these than in the normal tissues of origin. This is taken as a reflection of increased nuclear activity.

An absolute gain in the nuclear content of deoxyribonucleic acid (DNA) of neoplastic cells is commonly found, but less is known of the concomitant changes in other nuclear components, or of the relationship between nuclear and cytoplasmic metabolism.

Many of the parameters used in studies of cell metabolism in carcinoma and lymphoma cells have been shown to be ploidy dependent ; these include cell volume, nuclear volume and nucleic acid contents, together with those of several amino-acids and enzymes (Kit, 1960). In view of the proportional relationship between chromosome number and deoxyribonucleic acid content, the latter has frequently been used as an index of the level of ploidy. The DNA value taken to be equivalent to the diploid number of chromosomes is that of the so-called resting stage (interphase) and refers to the post-telophase level. However, synthesis of DNA occurs during a period of interphase when, preparatory to mitosis, it continues until the nuclear content is doubled. Subsequently the DNA content falls abruptly to the original level as the cell divides into two daughter cells, each new cell taking an equal share in a normal mitotic division. So whilst the chromosome number of a cell is constant for a given species without reference to the stage of the mitotic cycle, the DNA content undergoes a cyclical change.

When analyses of normal and neoplastic tissues are carried out by biochemical methods, the results may be expressed in terms of the average value only of a given substance per cell. Daoust (1958) has drawn attention, however, to the fallacies of this practice when carried out on a heterogeneous cell population without reference to possible changes in the numerical proportions of different cell types. The same objection may be held to apply to some extent to a relatively homogeneous population at various stages of the mitotic cycle when one of the parameters used for comparison is known to vary with this cycle yet no reference is made to the particular stage.

Cell metabolism in general might be expected to be more closely related to the stage of the mitotic cycle in a rapidly-dividing cell population than in one with a prolonged interphase, since in the former a larger proportion of the total cell metabolism is taken up with processes relating to division. Thus in the investigation of the quantitative relationship between various substances and the level of ploidy as shown by DNA values, it would seem that measurements at the level of individual cells rather than at that of whole cell populations would be a more sensitive guide, particularly in a rapidly dividing mass.

This paper describes the relationship between DNA value and the protein content as shown by the Naphthol Yellow-S technique of Deitch (1955), using microdensitometric measurements on individual cells.

### METHODS AND MATERIALS

Ascites tumour cells (T.2146), originally isolated from a tar-induced carcinoma of skin, were maintained in an inbred strain of white mice. Immediately after harvesting, smears were made on clean slides and fixed in absolute methyl alcohol. Each slide was then stained by the Feulgen technique and Naphthol Yellow-S according to the method of Deitch (1955) ; the use and validity of this approach is considered elsewhere (Meek, 1962). Microdensitometry was carried out on a scanning and integrating instrument with random selection of cells as in previous work (Meek, 1961*a*, 1961*b* ; Meek and Sparshott, 1961), using normal leucocytes as a readily available diploid standard for estimation of DNA content by absorption of the Feulgen stain. The technique has been discussed by Hale and Wilson (1961). Measurements were made on ascites tumour cells in interphase, recording the absorption of the Feulgen stain at 550 m$\mu$., and that of the Naphthol Yellow-S at 430 m$\mu$. Two recordings were made on each cell, one at the higher and one at the lower wavelength.

The results were plotted graphically so as to compare the values of the two components in single cells ; arbitrary units denote the values of DNA and protein.

### RESULTS

Examination of the graph (Fig. 1) shows there to be a positive correlation ($r = 0{\cdot}75$) between the DNA content of a tumour cell and the protein complement shown by this particular staining technique.

The distribution of DNA content of these cells during interphase lies mostly between near-tetraploid and octoploid values when using normal leucocytes as a standard of comparison, the lower value representing the post-telophase and the higher the pre-prophase levels.

The mean value of protein content in the near-tetraploid cells is approximately one half that in the near-octoploid cells. Therefore, both DNA and protein show a concurrent doubling of value from one end of the ploidy range to the other. There are, in addition, a small number of values recorded which correspond to much higher degrees of ploidy. In these latter, there is about the same ratio between the two series of readings as in the main group of cells. The same relationship therefore seems to hold good throughout the whole range.

At the same time it is seen that the scatter of values recorded as indicative of protein content against any given level of DNA content is very wide.

### DISCUSSION

Naphthol Yellow-S was introduced by Deitch (1955) who investigated the properties and use of this dye as a quantitative reagent for the estimation of protein. It is considered to react in a stoichiometric manner with the available

Fig. 1.—The readings represent total cellular protein plotted against readings for total cellular deoxyribonucleic acid. Both are in arbitrary units. In many cases, more than one set of readings falls on the same point ; such points are shown by larger solid black circles, which may represent two or more overlapping values.

ε-basic groups of lysine, arginine and histidine so that the recordings given here are a measure of the number of these groups available for binding of the dye. Deitch (1955) noted that the basic proteins bound to nucleic acids do not react unless the nucleic acid is removed either by trichloracetic acid or by enzymes.

No attempt was made here to release this type of protein—this is being investigated separately. The use of this dye as a measure of protein content must therefore be viewed in the light of these reactions and the conditions of application (Meek, 1962). Deitch (1955) compared the results obtained by its quantitative use with the values given on the same proteins by other authors using standard biochemical methods, and showed good correlation between the two series.

It is apparent from Fig. 1 that there is a trend associating low values of DNA with low values of protein, and high DNA values with high protein values. Since these represent cells in interphase chosen at random and taken from a rapidly proliferating population, it is highly probable that some are at the post-telophase stage and some at the pre-prophase stage. At first sight it would seem, therefore, that the protein content is ploidy-dependent throughout the cycle. Before this can be accepted, however, the question of heterogeneity of the population and its effect on these readings must be considered.

Although it appears to be true in general that the growth of a tumour depends mainly on the proliferation of its stemline cells, it is likely that some degree of genetic variation is present in all neoplasms. However, a study of the distribution of values of DNA content has already been carried out on these cells during interphase and other stages of the mitotic cycle (Meek, 1961*a*) showing that the great majority of cells which successfully complete mitosis are represented by a particular value of DNA corresponding to the stem-cell content. In Fig. 1 this value falls at the lower end of the range of DNA measurements. It is concluded from this evidence that, whilst the lower DNA readings probably represent mostly stemline cells at the post-telophase level, the higher readings include an appreciable number of stemline cells at other stages of interphase.

If it is accepted, therefore, that the range of DNA measurements shown here represents a fair distribution of cells at different stages of interphase, then it seems that the protein content is quantitatively associated with the level of cell ploidy at all stages of the mitotic cycle. Thus the average reading for the protein complement corresponding to a low level of DNA-ploidy is one half of the average reading found at twice that level of ploidy. At the same time, it is clear that there is a wide variation of protein content for all degrees of ploidy, so much so that some protein measurements recorded for tetraploid cells are equal to those from octoploid cells. Both the trend and the variation need to be considered.

A positive correlation between protein content and DNA complement places the results in the same class as those obtained for a number of other parameters (Kit, 1960), though not all cell components investigated from this point of view have been found to be quantitatively linked to the degree of ploidy. However, whilst these findings appear to fall parallel with those of other workers, and to provide another example of partial control of metabolism by gene-dosage effect as discussed by Kit (1960), there is an essential difference. Other studies have been based on the level of ploidy determined either by the chromosome number of the stemline, or by the average DNA content. In the latter case, a single value is used which takes no account of the doubling of DNA between post-telophase and pre-phophase stages, so that whilst it is evident that a given cell constituent has twice the average value in tetraploid as in diploid cells, it is not clear whether its value is also increased proportionately to that of DNA during synthesis of the latter in preparation for cell division. In the present study the trend of association suggests that the pre-mitotic increase of DNA content is linked with a proportionate rise in the cellular complement of protein ; in effect, ploidy-dependence holds throughout the mitotic cycle.

As to the wide scatter of results, it may not be expected that there would be close quantitative dependence between a metabolically active substance and the basic genetic material since only a minor percentage of DNA is in an active form (Danielli, 1961). The wide variation seen at all levels may be due in part to errors of measurement and also to the presence of cells, other than those of the stemline, showing quantitative relationships between DNA and protein content basically different from that of the stemline and from one another. These are in a minority, however, and the variations are considered to be due largely to differences in the metabolic states of individual cells.

Whilst it may be agreed that not all of the basic content is in an active form, it is not clear whether all newly-synthesised DNA is entirely inactive until the time of the succeeding cell division, or whether a proportion corresponding to that of the original complement of DNA immediately becomes active so far as its effect on general cell metabolism goes. Although these results offer no proof that newly synthesised DNA is active in this sense, they can be held in favour of this view.

Against this it may be argued that these are undifferentiated cells dividing rapidly, and that their interphase cell metabolism is linked almost wholly to processes concerned with preparation for cell division. If so, the protein measured by this technique may indicate substances synthesised in step with DNA. Studies on cells with a low mitotic rate and a long interphase in which DNA synthesis occupies a lesser proportion of the total interphase activity, would throw further light on this. The basic protein linked directly to nucleic acid has not been considered in this investigation.

The quantitative dependence of certain cell components on the level of ploidy can only be considered in general terms, since it is clear that many transient conditions are capable of influencing cell metabolism. The interpretation favoured here is that the level of ploidy is related to certain limits within which quantitative changes in some cell substances can take place according to the effect of other factors. If it is accepted that DNA is basically responsible for cell metabolism in general, then these limits can be said to be set by the level of DNA content.

### SUMMARY

Measurements were made by microdensitometry on mouse ascites tumour cells in interphase. The total cell content of protein as shown by the method of Deitch (1955), was related to the total content of deoxyribonucleic acid of the same individual cells.

The results showed a trend of association linking the total cellular protein to the level of cell ploidy. The scatter of results was thought to reflect the influence of other factors at all levels. In addition, it was considered that DNA newly-synthesised during the mitotic cycle may have an immediate effect on the general level of cell metabolism.

I wish to thank Professor T. F. Hewer for reading this manuscript, Miss Joan Little for technical assistance, Mr. Dennis White for photography, and the British Empire Cancer Campaign for full financial assistance.

### REFERENCES

DANIELLI, J. F.—(1961) Proceedings of the Conference on Biochemistry of Human Cancer. Chester Beatty Research Institute, May, 1961. p. 9.
DAOUST, R.—(1958) Symposium on Liver Function, Publ. No. 4, Amer. Inst. of Biol. Sci., Washington, D.C., p. 3.
DEITCH, ARLINE, D.—(1955) *Lab. Invest.*, **4**, 324.
HALE, A. J. and WILSON, SYLVIA J.—(1961) *J. Path. Bact.*, **82**, 483.
KIT, S.—(1960) *Cancer Res.*, **20**, 1121.
MEEK, E. S.—(1961*a*) *Brit. J. Cancer*, **15**, 162.—(1961*b*) *J. Path. Bact.*, **82**, 167.—(1962) *J. Histochem. Cytochem.* (In press).
*Idem* AND SPARSHOTT, Sheila, M.—(1961) *Naturwissenschaften*, **48**, 726.